# Cross-cultural adaptation of the Arabic version of the oral health values scale

**DOI:** 10.2340/aos.v83.41092

**Published:** 2024-08-26

**Authors:** Salema Traina, Daniel W. McNeil, Maha El Tantawi, Arheiam Arheiam

**Affiliations:** a Department of Dental Public Health and Preventive Dentistry, Faculty of Dentistry, University of Benghazi, Benghazi, Libya; b DN-COMMUNITY DENTISTRY, University of Florida, Gainesville, FL, USA; c Department of Dental Public Health and Preventive Dentistry, Faculty of Dentistry, Alexandria University, Alexandria, Egypt

**Keywords:** Cross-cultural adaptation, oral health value, reliability, validity

## Abstract

**Aims:**

The study aimed to adapt the original English-language oral health values scale (OHVS) to the Arabic culture and to test its psychometric properties.

**Methods:**

The original OHVS was translated into Arabic language and pre-tested using cognitive interviewing. The psychometric properties of Arabic OHVS were examined in a sample comprising 416 Libyan adults aged 18–70 years, recruited from the main public and private dental clinics in Benghazi. The participants’ demographic information, oral health behaviour, perceived oral health, the Arabic version of the OHVS (A-OHVS), dental neglect scale, oral health-related quality of life, oral health literacy, simplified oral hygiene index (OHI-S), and decayed, missing, and filled teeth index (DMFT) were collected. Psychometric properties were tested using content validity, construct validity, discriminating validity, internal consistency, test–retest reliability, and floor as well as ceiling effects were examined.

**Results:**

The Arabic OHVS was successfully and smoothly developed. It showed an acceptable level of equivalence to the original version, The A-OHVS presented an overall Cronbach’s Alpha of 0.74 and the average score was 40.02, ranging between 26 and 54. All hypotheses predefined to test construct validity were confirmed. The bivariate correlation between A-OHVS and other health indicators shows a significant positive correlation between A-OHVS and oral health literacy (*p* = 0.000). On the other hand, statistically significant negative correlations were observed between A-OHVS and dental neglect and quality of life (*p* ≤ 0.001) as well as DMFT and OHI-S (*p* ≤ 0.001). Floor or ceiling effects were not observed.

**Conclusions:**

The A-OHVS was shown to be a valid and reliable tool for assessing oral health values in the Arabic-speaking population.

## Introduction

Oral health is no longer seen as merely the absence of disease and infirmity. Oral health is recognized as an integral component of physical, mental, and social well-being and quality of life [1]. This concept of oral health has been translated into a definition developed by the World Dental Federation (FDI) [1], which envisions oral health as a multifaceted construct, with multiple attributes, existing on a continuum, and is influenced by an individual’s attitudes, values, perceptions, and experiences [2]. In addition, the FDI has developed a companion framework that includes three core elements (disease and condition status, physiological function, and psychosocial function) and sets of driving and moderation factors. Consistent with this definition of oral health, a recent initiative emphasized the importance of integrating behavioural and social determinants in oral health assessment [3]. Accounting for an individual’s values, perceptions, and expectations is at the centre of the new concept of oral health. Personal values are a strong moderating factor that is correlated with health and well-being [4, 5], through influencing health behaviour [6]. Many health behaviour theories have acknowledged the role that values play in shaping health behaviours [7–10], to the extent that is sufficient to induce or maintain a health-related behaviour [11].

While several measures and socio-dental indicators have been developed to assess oral health, and patients’ needs and outcomes [12, 13], little work has been conducted on measuring and evaluating oral health values (OHV) which can be defined as the extent to which one views dental status as essential or worthwhile by dedication to improving or maintaining one’s teeth, gingiva, and other aspects of orofacial functioning [14]. Studying OHV may enable researchers to better understand the psychosocial barriers and disparities of dental care utilization. Therefore, a new oral health values scale (OHVS) has been developed and validated in the United States [14]. The developed tool comprised 12 items and four subscales (Professional Dental Care, Appearance and Health, Flossing and Retaining Natural Teeth). The OHVS has been translated into Portuguese and Romanian languages using a population-based survey of Portuguese adults [15] and an online survey of Romanian adults [16].

So far, there has not been any attempt to develop an Arabic version of this instrument. In addition, none of the studies used clinical indicators to validate OHVS. It is recommended to cross-culturally adapt and test the psychometric properties of self-report measures before they can be used in a different context or cultural group to ensure their suitability to the new culture and their equivalence to the original measure [17–19]. Thus, the aim of this study is to develop the Arabic version of OHVS (A-OHVS) and to test its validity using clinical assessment.

## Methods

The sequence and procedures used for the cross-cultural adaptation and psychometric testing of the Arabic OHVS followed the guidelines proposed by Beaton et al. [17]. The guideline proposed included three main stages: (1) Translation of the original tool (Two versions of forward and backward translations were developed and discussed by four independent translators and reviewed by an expert committee comprising the developer, a professional language expert, and a methodologist). (2) Testing the pre-final version of the translated tool among 30–40 individuals. (3) Testing the psychometric and measurement properties among larger populations [17]. Ethical clearance and permissions for the study were obtained from the Faculty of Dentistry at the University of Benghazi, Libya prior to data collection (Ref: Ben-Dent-056). Verbal informed consent was obtained from participants.

### Stage 1: Translation of the original OHVS

The aim of the translation stage is to develop a pre-final A-OHVS which is conceptual and semantic equivalence to the original English language OHVS (OV). The OV was translated into Arabic using a rigorous forward–backwards translation process. The OV was first translated into Arabic by two bilingual native Arabic speakers who performed the translations independently and were aware of the aim of the study. The created Arabic translations (T1 &T2) were then discussed among a team of four researchers to merge them into one Arabic version (T12). A back translation of the Arabic version (T12) into English language was then conducted by two other bilingual English and Arabic speakers who created two independent back-translations (BT1 & BT2) which were then discussed with the investigators to generate one English version (BT12). The expert committee, including the developers of OV, reviewed the translations and assessed their equivalence to the OV [18] to approve a pre-final version of the A-OHVS. No major or meaning-related modifications were recommended.

### Stage 2: Testing of pre-final A-OHVS

The aim of this stage was to test the face and content validity of the translated OV. The pre-final A-OHVS was sent to 11 oral health experts, academics, and practitioners from different Arabic countries to test face and content validity of the A-OHVS. The experts were asked to rate and provide feedback regarding the translated items of the scale for relevance and representativeness of the construct of oral health value and also to rate each item for specificity and clarity of wording for each item, using a five-point Likert scale ranging from strongly disagrees to strongly agree. Overall, the items were rated as relevant, clear, and specific in their meaning.

The pre-final A-OHVS was also tested among a group of 40 Arabic-speaking adults (Libyan and non-Libyans) who were dental patients in one public dental clinic, schoolteachers and administrative and non-dental workers at the University of Benghazi. The participants were asked to complete the A-OHVS and to provide feedback regarding each item in terms of the clarity of meaning and wording, relevance, and difficulty to answer.

### Stage 3: Psychometric properties of A-OHVS

This stage was conducted to assess measurement and psychometric properties of the developed tool among wider population. A cross-sectional survey was used to examine the psychometric properties of the A-OHVS in a sample of Libyan adults aged 18–70 years. A minimum sample size of 400 has previously been identified to be sufficient for studies assessing reliability and validity [20]. A convenience sampling strategy was employed to recruit the study participants from dental practices in the public and private sectors in Benghazi. The potential participants were approached in the waiting rooms, the aims of the study were explained, and verbal consent was obtained. Both a self-administered questionnaire and clinical examination were used for data collection.

### Questionnaire

A self-administered questionnaire was handed out to the participants by the principal investigator who was available for assistance and to answer any queries. The questionnaire took a maximum of 20 min to complete and comprised five sections. Section 1 assessed socio-demographic information, and included age, gender, education (primary, secondary, university, postgraduate). Section 2 assessed oral health behaviours and self-reported oral health including use of dental floss, frequency of toothbrushing (never, sometimes, once a day, twice a day) and the reason for dental visit (emergency, checkup, and treatment), and a question on patient’s self-reported oral health where they select one score on a 5-point Likert scale ranging from poor to excellent. Section 3 included A-OHVS, Arabic versions of dental neglect scale (DNS), oral health literacy scale (OHL), and oral health impact profile (OHIP-5). DNS is a six-item Likert-type scale. It assesses the extent to which individuals care for their dental health, go to the dentist, and value their oral health [21]. OHIP-5 is the shortest version of OHIP which aims to capture impacts related to oral function, orofacial pain, orofacial appearance, and psychosocial impact and has been conceptualised as adverse outcomes [22]. OHL was assessed using the short of Health literacy in Dentistry (HeLD-14) [23]. The A-OHVS was administered again after 3 weeks to a randomly selected sub-sample of 50 participants. This step was undertaken to allow the assessment of test–retest reproducibility.

### Clinical examination

Three experienced dentists were trained and calibrated to perform clinical dental examinations. Intra-examiner reliability and inter-examiner reliability were tested in a separate group of Libyan adults before commencing the data collection of the main study. Kappa coefficients ranged from 0.87 to 0.93. All dental examinations were conducted while participants were seated on a dental chair, using a disposable mouth mirror and probe and artificial dental chair light. Decayed, missing, and filled teeth index (DMFT) was used to measure caries experience according to WHO criteria [24]. The simplified oral hygiene index (OHI-S) was used to measure oral hygiene which was then classified into good (score ≤ 2), fair (score = 2.1–4), and poor (score = 4.1–6) [25]

### Data analysis

Data analyses were conducted using SPSS software (IBM, Version 25). Descriptive statistics were used to describe the sample profile and the presence of ceiling or floor effects by calculating the frequencies of the lowest or highest possible scores to assess whether they exceed 15% of total responses [26].

Internal consistency was assessed by calculating Cronbach’s alpha coefficient for the overall scale and each subscale. Cronbach’s alpha values ≥ 0.6 were considered an acceptable level [27]. Intra-class correlation coefficient (ICC) was used to assess test–retest reliability and was calculated from the repeated administrations of the questionnaire. An ICC of 0.7 indicates an acceptable level of reproducibility [28].

Discriminate validity [29] was assessed against predefined hypotheses [26], as follows: lower A-OHVS scores would be observed among those who (1) had less than 20 natural teeth; (2) had active caries at dentin level (more than one decayed tooth vs. caries-free); (3) had poorer oral hygiene according to OHI-S; (4) reported irregular brushing or never brushed their teeth; (5) visited the dentist because of dental pain, and; (6) had poor perception of their oral health defined based on dichotomization of responses (good-excellent vs. fair-bad). In addition, it postulated that A-OHVS was positively correlated with HeLD14 scores and negatively correlated with A-DNS and OHIP-5. All hypotheses were tested by conducting an independent sample t-test and one-way ANOVA test to compare groups and correlations were tested using Pearson’s correlation test. An exploratory factor analysis (EFA) was conducted to test the factorial validity of items in the subscales defined in the original OHVS, using the varimax rotation and a strict cut-off of factor loading of > 0.40 [30]. All analyses were carried out at *p* < 0.05.

## Results

### Face and content validity

All items were considered relevant and clearly understood. No changes were suggested in relation to questionnaire items, response options, or mode of self-administration. The final A-OHVS was cross-culturally adapted with acceptable content validity and face validity.

### Psychometric properties

A total 500 participants were invited to take part in the study and 416 were included in the analysis (response rate 83%) (Table 1 shows the socio-demographic characteristics of the study sample). Female participants were more than males (246, 59.9%), attended state-run dental facilities (268, 64.4%), and had a university degree (60.1%), with an average age of 37 years (SD = 12). Most participants (61.1%) reported brushing their teeth twice per day. Although the majority knew about dental floss (71.9%), most of them did not use it (72.4%). The participants visited the dentists, mainly because of dental pain (27%) and receiving treatments such as scaling (16%) and filling (14%). Tables 2 and 3 show the minimum, maximum, and average scores, ICC and Cronbach’s Alpha for A-OHVS, and its subscales. Overall Cronbach’s Alpha was 0.72 and the average score was 41.02, ranging between 26 and 54. The lowest value for Cronbach’s Alpha was for the professional care subscale (0.61) whereas the highest value was for the flossing subscale (0.77). ICC for A-OHVS was 0.94 and the ICC of the subscales ranged between 0.81 and 0.91. The frequencies of the lowest and highest scores were 1 (0.2%).

**Table 1:** Distribution of sociodemographic characteristics and oral health behaviours of the study sample (*n* = 416).

Variable		Count	%
Gender	Male	167	40.1
	Female	249	59.9
Clinic	Private	148	35.6
	Public	268	64.4
Education level	Primary or less	19	4.6
	Preparatory/secondary	128	30.8
	University	269	64.7
	Postgraduate	19	4.6
Times per day for toothbrushing	Twice	254	61.1
	Once	109	26.2
	Sometimes	24	5.8
	Never	29	7.0
Knowledge about dental floss	Yes	299	71.9
	No	117	28.1
Times per day of dental floss use	Twice	5	1.2
	Once	27	6.5
	Sometimes	83	20.0
	Never	301	72.4
Reason for the last dental visit	Pain	189	45.4
	Check-ups	114	27.4
	Follow-up and treatment	113	27.2
Caries	No caries	59	14.2
	Have caries	357	85.8
Natural teeth	Has 20 teeth or more	390	93.8
	Has less than 20 teeth	26	6.3
Oral hygiene according to OHI-S	Good	186	44.7
	Fair	182	43.8
	Poor	48	11.5

OHI-S, simplified oral hygiene index.

**Table 2:** Average, minimum, and maximum scores and Cronbach’s Alpha for A-OHVS scale and subscales among the study sample.

OHVS subscale	Mean	SD	Min	Max	Cronbach’s Alpha	Intra-class correlation
Professional Dental Care	8.05	2.79	3	14	0.61	0.91
Appearance and Health	14.00	1.34	8	15	0.63	0.81
Flossing	5.96	2.68	3	13	0.74	0.89
Retaining Natural Teeth	12.78	2.24	6	15	0.62	0.84
Overall	41.02	5.25	26	54	0.72	0.94

OHVS, oral health values scale; A-OHVS, Arabic version of OHVS.

**Table 3:** Average scores, corrected items correlations, and Cronbach’s Alpha if Item Deleted for AOHVS and subscales.

Item	Mean	SD	Corrected-item correlations	Scale mean if item deleted	Cronbach’s alpha if item deleted	Cronbach’s alpha for subscale if item deleted
OHVS1	4.69	0.53	0.31	36.34	0.71	0.74
OHVS2	1.84	1.02	0.47	39.19	0.68	0.65
OHVS3	4.79	0.53	0.31	36.24	0.71	0.60
OHVS4	2.02	0.97	0.30	39.01	0.71	0.76
OHVS5	2.09	1.10	0.41	38.94	0.70	0.55
OHVS6	4.34	0.80	0.18	36.69	0.72	0.21
OHVS7	4.53	0.71	0.46	36.50	0.69	0.46
OHVS8	2.83	1.43	0.55	38.19	0.68	0.12
OHVS9	3.76	1.44	0.12	38.05	0.73	0.26
OHVS10	2.04	1.17	0.57	38.99	0.66	0.77
OHVS11	3.21	1.30	0.40	36.83	0.69	0.36
OHVS12	4.69	0.52	0.21	36.33	0.72	0.49

OHVS, oral health values scale.

Table 4 shows comparisons of mean scores of A-OHVS according to the presence of caries, having less than 20 natural teeth, frequency of toothbrushing, reason for dental visit, and perceived oral health. The mean score of the overall A-OHVS scale was significantly higher among those who rated their oral health as ‘good/excellent’ (*p* ≤ 0.001), had no dental caries (*p* < 0.001), had more than 20 natural teeth (*p* ≤ 0.001), visited the dental clinic for a check-up (*p* < 0.001), and regularly brushed their teeth on a daily basis (*p* < 0.001).

**Table 4:** Comparisons of A-OHVS scores according to clinic type, caries, number of natural teeth, frequency of toothbrushing, and perceived oral health and reason for dental visit.

Variables		Total A-OHVS		*p*
		Mean	SD	
Dental caries	No caries	42.93	4.95	< 0.001
	Have caries	40.71	5.25	
Natural teeth	Has 20 teeth or more	41.29	5.16	< 0.001
	Has less than 20 teeth	37.08	5.16	
Oral hygiene according to OHI-S	Good	42.07	4.81	< 0.001
	Fair	40.69	5.44	
	Poor	38.22	5.12	
Toothbrushing	Regular	41.29	5.28	< 0.001
	Irregular	39.23	4.78	
Perceived oral health	Fair to Bad	40.48	4.85	< 0.001
	Good to excellence	42.03	5.81	
Dental visit	check-up	42.78	5.63	< 0.001
	Pain	40.12	4.81	
	Treatment and follow up	40.78	5.20	

OHVS, oral health values scale; OHI-S, simplified oral hygiene index; A-OHVS, Arabic version of OHVS.

(*t* test and ANOVA were used for groups comparisons).

Table 5 shows the correlation between OHVS and HeLD14, DNS, OHIP-5, and OHI-S. A statistically significant positive correlation was observed between OHVS and HeLD14 (*p* ≤ 0.001). On the other hand, statistically significant negative correlations were observed between OHVS and DNS, OHIP-5, and OHIS (*p* ≤ 0.001).

**Table 5:** Pearson’s r correlation coefficient between A-OHVS and the scores of various health indicators.

Health indicators and scales	Correlation coefficient	*p*
OHIP-5	-0.221^**^	< 0.001
DNS	-0.370^**^	< 0.001
HeLD14	0.490^**^	< 0.001
OHI-S	-0.240^**^	< 0.001

OHIP-5: Oral health impact profile (five items); DNS: dental neglect scale; HeLD14: health literacy of dentistry (14 items); OHI-S: simplified oral hygiene index; A-OHVS, Arabic version of OHVS.

Pearson correlation was used to test correlations.

Table 6 presents the EFA and item-impact analysis. The EFA returned a 4-factor solution which explained 59% of the variance. There were no changes in items for flossing (#2,5,10) and appearance and health sub-scales (#3,7,12). There were changes in items previously loaded on natural teeth’ and professional care factors. The item ‘OHVS1’ loaded into professional and dental care subscale along with items (#8 &11). The item ‘OHVS4’ loaded into natural teeth subscale, along with items (#6&9).

**Table 6:** Factor loadings of A-OHVS based on EFA with Varimax rotation.

Items		Factors			
		1	2	3	4
OHVS1	It is important to me to keep my natural teeth.*	0.790			
OHVS2	It is okay for me to miss a day or two of flossing when I am busy			0.850	
OHVS3	My smile is an important part of my appearance.		0.561		
OHVS4	Going to a dentist is not worth the cost to me.*				0.607
OHVS5	Flossing my teeth every day is a high priority for me			0.858	
OHVS6	I would rather get dentures than spend money to treat cavities or gum disease				0.674
OHVS7	I think it is important that my teeth and gums are a source of pride.		0.726		
OHVS8	If I have a toothache, I prefer to wait and see if it will go away on its own before seeing a dentist	0.490			
OHVS9	I would not mind if I had to have a false tooth or dentures.				0.681
OHVS10	I make sure I have dental floss available with me so I have it when I need it			0.627	
OHVS11	Going to the dentist is only important if my teeth or gums are bothering me.	0.459			
OHVS12	The condition of my teeth and gums is an important part of my overall health.		0.837		

OHVS, oral health values scale; A-OHVS, Arabic version of OHVS; EFA, exploratory factor analysis.

Note: factor 1 = professional and dental care; factor 2 = appearances and health; factor 3 = flossing factor; factor 4 = retaining natural teeth.

* indicates items that were loaded on different factors.

## Discussion

Oral health values have received much attention in behavioural dentistry as a social determinant of oral health by influencing important behaviours such as seeking dental care [6]. The present study cross-culturally adapted the A-OHVS. The developed Arabic version was found to be equivalent to the original English version. The translation’s acceptability was confirmed by experts and participants from the general population. The A-OHVS had good internal consistency and test–retest reliability. It discriminated between participants based on oral health behaviours, self-reported and clinically assessed oral health status. It had good construct validity in relation to the DNS, HeLD14, OHIP-5, and OHIS. In addition, the EFA retained the 4-factor structure of A-OHVS but showed different subscale loading for the professional care and maintaining natural dentition factors. Flossing and health and appearance factors remained stable which may explain their high internal consistency.

The study had some limitations. There is a potential for social desirability and recall biases that are inherent in studies based on self-reporting. Also, participants were recruited from hospitals only and further studies including the general population are advisable. Finally, the study was conducted in one Arabic country. The study had several strengths, nevertheless. Experts and participants from various Arabic countries contributed to the pre-testing and validation of the A-OHVS. The questionnaire was translated into formal Arabic that is understood by all Arab-speaking populations and is used as the official language in education. This formal Arabic is understood by different Arabic nations regardless of their regional dialects. An important strength of the study is using the objective, clinically assessed oral health status indicators to validate the A-OHVS.

The psychometric testing of A-OHVS showed an acceptable overall Cronbach’s alpha [31]. Higher Cronbach’s alpha values have been reported in the original, Portuguese, and Romanian versions of OHVS [15, 16]. Different factors reduce the value of Cronbach’s alpha such as the small number of items and multiple subdomains [32]although these factors were similar in the A-OHVS and the original OHVS. Further studies are needed to confirm these differences and elucidate the reasons for them. The ICC scores for the overall A-OHVS and its subscales were satisfactory and comparable to those in previous studies of OHVS validation [15, 16]. Their values were above the recommended threshold [33], indicating very good reproducibility [28].

The average score of A-OHVS in the present study was lower than that reported among Portuguese or Romanian participants. The reduced average score in the A-OHVS may have something to do with cultural differences in conceptualizing and viewing OHV as a construct or may be related to the characteristics of the study participants. For example, the study participants had a relatively low average age that might have affected their perception of the importance and relevance of the items related to maintaining natural dentition and professional care in comparison to health and appearance items.

The A-OHVS demonstrated good discriminate validity. Statistically higher scores of A-OHVS were reported among participants who were caries-free, with natural dentition, good oral hygiene according to OHI-S, regularly brushed their teeth, visited the dentist for check-ups, and considered their oral health as good-excellent. Taken together, these findings support the theory that those who value their oral health tend to display better oral health behaviours and outcomes [6]. Likewise, the A-OHVS displayed high convergent validity with HeLD14, DNS, and OHIP-5 that examine the perception of oral health and was expected to converge with OHV [14, 13, 34]. Higher A-OHVS was correlated with higher HeLD14 scores, lower dental neglect, and quality of life impacts. Similar findings have been observed in previous studies of OHVS. These findings confirm the construct validity of the A-OHVS and support the notion that OHVs play a role in predicting oral health outcomes, oral health behaviours including the utilization of dental services. It is, therefore, useful to use A-OHVS in future studies to explain disparities in oral health behaviours and outcomes and to understand health seeking behaviours among Arabic speaking adults.

## Conclusions

The A-OHVS has been successfully adapted to the Arabic language. The A-OHVS has demonstrated acceptability to excellent internal consistency, test re-test reliability, discriminant and construct validity as a tool for measuring OHV that can be used in future research and assessment of oral health risk factors.

## Supplementary Material

Cross-cultural adaptation of the Arabic version of the oral health values scale

## References

[1] Hescot P. The new definition of oral health and relationship between oral health and quality of life. Chin J Dent Res. 2017;20(4):189–192. 29181455 10.3290/j.cjdr.a39217

[2] Glick M, Williams DM, Kleinman DV, Vujicic M, Watt RG, Weyant RJ. A new definition for oral health developed by the FDI World Dental Federation opens the door to a universal definition of oral health. J Am Dent Assoc (1939). 2016;147(12):915–917. 10.1016/j.adaj.2016.10.001 27886668

[3] McNeil DW, Randall CL, Baker S, et al. Consensus statement on future directions for the behavioral and social sciences in oral health. J Dent Res. 2022;101(6):619–622. 10.1177/00220345211068033 35043742 PMC9124906

[4] Sagiv L, Schwartz SH. Value priorities and subjective well-being: Direct relations and congruity effects. Eur J Soc Psychol. 2000;30(2):177–198. 10.1002/(SICI)1099-0992(200003/04)30:2<177::AID-EJSP982>3.0.CO;2-Z

[5] Schwartz SH, Cieciuch J, Vecchione M, et al. Refining the theory of basic individual values. J Pers Soc Psychol. 2012;103(4):663. 10.1037/a0029393 22823292

[6] Fisher-Owens SA, Gansky SA, Platt LJ, et al. Influences on children’s oral health: a conceptual model. Pediatrics. 2007;120(3):e510–e520. 10.1542/peds.2006-3084 17766495

[7] Patrick DL, Lee RSY, Nucci M, Grembowski D, Jolles CZ, Milgrom P. Reducing oral health disparities: a focus on social and cultural determinants. BMC Oral Health. 2006;6(1):S4. 10.1186/1472-6831-6-S1-S4 PMC2147600 16934121

[8] Ajzen I. The theory of planned behavior. Organ Behav Hum Decision Process. 1991;50(2):179–211. 10.1016/0749-5978(91)90020-T

[9] Behling O, Starke FA. The postulates of expectancy theory. Acad Manage J. 1973;16(3):373–388. 10.2307/254999

[10] McNeil DW. Behavioural and cognitive-behavioural theories in oral health research: current state and future directions. Community Dent Oral Epidemiol. 2023;51(1):6–16. 10.1111/cdoe.12840 36779644 PMC10516240

[11] Cooke R, French DP. How well do the theory of reasoned action and theory of planned behaviour predict intentions and attendance at screening programmes? A meta-analysis. Psychol Health. 2008;23(7):745–765. 10.1080/08870440701544437 25160879

[12] Sheiham A, Tsakos G. Oral health needs assessment. New Malden: Quintessence; 2007.

[13] Sischo L, Broder HL. Oral health-related quality of life: what, why, how, and future implications. J Dent Res. 2011;90(11):1264–1270. 10.1177/0022034511399918 21422477 PMC3318061

[14] Edwards CB, Randall CL, McNeil DW. Development and validation of the oral health values scale. Community Dent Oral Epidemiol. 2021;49(5):454–463. 10.1111/cdoe.12621 33734475 PMC8518540

[15] Machado V, Mendonça A, Proença L, et al. Cross-cultural adaptation and validation of the oral health values scale for the Portuguese population. J Pers Med. 2022;12(5):672. 10.3390/jpm12050672 35629094 PMC9143491

[16] Balgiu BA, Sfeatcu R, Mihai C, Lupușoru M, Bucur MV, Tribus L. Romanian version of the oral health values scale: adaptation and validation. Medicina. 2022;58(4):544. 10.3390/medicina58040544 35454382 PMC9031385

[17] Beaton DE, Bombardier C, Guillemin F, Ferraz MB. Guidelines for the process of cross-cultural adaptation of self-report measures. Spine. 2000;25(24):3186–3191. 10.1097/00007632-200012150-00014 11124735

[18] Herdman M, Fox-Rushby J, Badia X. A model of equivalence in the cultural adaptation of HRQoL instruments: the universalist approach. Qual Life Res. 1998;7(4):323–335. 10.1023/A:1008846618880 9610216

[19] Guillemin F, Bombardier C, Beaton D. Cross-cultural adaptation of health-related quality of life measures: literature review and proposed guidelines. J Clin Epidemiol. 1993;46(12):1417–1432. 10.1016/0895-4356(93)90142-N 8263569

[20] Charter RA. Sample size requirements for precise estimates of reliability, generalizability, and validity coefficients. J Clin Exp Neuropsychol. 1999;21(4):559–566. 10.1076/jcen.21.4.559.889 10550813

[21] Coolidge T, Heima M, Johnson EK, Weinstein P. The dental neglect scale in adolescents. BMC Oral Health. 2009;9(1):1–7. 10.1186/1472-6831-9-2 19123953 PMC2627830

[22] Alhajj MN, Halboub E, Khalifa N, et al. Translation and validation of the Arabic version of the 5-item Oral health impact profile: OHIP5-Ar. Health Qual Life Outcomes. 2018;16(1):1–11. 10.1186/s12955-018-1046-0 30453965 PMC6245614

[23] Jones K, Brennan D, Parker E, Jamieson L. Development of a short-form Health Literacy Dental Scale (HeLD-14). Community Dent Oral Epidemiol. 2015;43(2):143–151. 10.1111/cdoe.12133 25388410

[24] World Health Organization. Oral health surveys: basic methods. World Health Organization; 2013. Geneva, Switzerland.

[25] Greene JG, Vermillion JR. The simplified oral hygiene index. J Am Dent Assoc. 1964;68(1):7–13. 10.14219/jada.archive.1964.0034 14076341

[26] Terwee CB, Bot SDM, de Boer MR, et al. Quality criteria were proposed for measurement properties of health status questionnaires. J Clin Epidemiol. 2007;60(1):34–42. 10.1016/j.jclinepi.2006.03.012 17161752

[27] Locker D, Slade G. Oral health and the quality of life among older adults: the oral health impact profile. J Can Dent Assoc. 1993;59(10):830–833, 7–8, 44. 8221283

[28] Gilchrist F, Rodd H, Deery C, Marshman Z. Assessment of the quality of measures of child oral health-related quality of life. BMC Oral Health. 2014;14(1):40. 10.1186/1472-6831-14-40 24758535 PMC4021173

[29] Broder HL, Wilson-Genderson M, Sischo L. Reliability and validity testing for the Child Oral Health Impact Profile-Reduced (COHIP-SF 19). J Public Health Dent. 2012;72(4):302–312. 10.1111/j.1752-7325.2012.00338.x 22536873 PMC3425735

[30] Osborne JW, Costello AB. Best practices in exploratory factor analysis: four recommendations for getting the most from your analysis. Pan-Pacific Manage Rev. 2009;12(2):131–146.

[31] Hajjar SJIJoQ, Methods QR. Statistical analysis: internal-consistency reliability and construct validity. Int J Quant Qual Res Methods. 2018;6(1):27–38.

[32] Tavakol M, Dennick R. Making sense of Cronbach’s alpha. Int J Med Educ. 2011;2:53–55. 10.5116/ijme.4dfb.8dfd 28029643 PMC4205511

[33] Nunnally JC, Bernstein I. The assessment of reliability. Psychometr Theor. 1994;3(1):248–292.

[34] Shea JA, Guerra CE, Ravenell KL, McDonald VJ, Henry CA, Asch DA. Health literacy weakly but consistently predicts primary care patient dissatisfaction. Int J Qual Health Care. 2007;19(1):45–9. 10.1093/intqhc/mzl068 17178765

