# Supplementary material for: Cross-cultural adaptation of the Arabic version of the oral health values scale
Source: Acta Odontol Scand. 2024 Aug 26;83:41092. doi: 10.2340/aos.v83.41092 (PMC11407107; doi:10.2340/aos.v83.41092)
Supplement: Cross-cultural adaptation of the Arabic version of the oral health values scale [file AOS-83-41092-s1.pdf]

Supplementary material has been published as submitted. It has not been copyedited or typeset by Acta Odontologica Scandinavica.

Original English OHVS	Arabic OHVS
1. It is important to me to keep my natural teeth.	1. الحفاظ على جميع أسناني الطبيعية يعتبرمهم بالنسبة لي
2. It is okay for me to miss a day or two of flossing when I am busy	2. لا بأس من تفويت يوم أو يومين من تنظيف الأسنان بالخيط تنظيف الأسنان, إذا كنت مشغولاً
3. My smile is an important part of my appearance.	3. إبتسامتي جزء مهم من مظهري.
4. Going to a dentist is not worth the cost to me.	4. الذهاب إلى طبيب الأسنان لا يستحق قيمته المالية بالنسبة لي.
5. Flossing my teeth every day is a high priority for me	5. تنظيف أسنانك بالخيط كل يوم يعتبر اولوية بالنسبة لي
6. I would rather get dentures than spend money to treat cavities or gum disease	6. أفضل أن يكون لدي طاقم أسنان صناعي عن دفع تكاليف الإهتمام بأسناني الطبيعيه.
7. I think it is important that my teeth and gums are a source of pride.	7. أعتقد أنه من المهم أن تكون أسناني ولثتي مصدر إعتزاز لي.
8. If I have a toothache, I prefer to wait and see if it will go away on its own before seeing a dentist	8. إذا كنت اعاني من ألم في الأسنان فمن الأفضل الانتظار ومعرفة ما إذا كان سيختفي من تلقاء نفسه قبل زيارة طبيب الاسنان.
9. I would not mind if I had to have a false tooth or dentures.	9. لا يزعجني إذا كان يجب أن يكون لديّ سن صناعية أو "جسر".
10. I make sure I have dental floss available with me so I have it when I need it	10. أتاكد ان خيط تنظيف الأسنان موجود دائما معي ليكون متوفر عندما احتاجه.
11. Going to the dentist is only important if my teeth or gums are bothering me.	11. أنا اعتقد ان زياره طبيب الاسنان مهمة فقط عندما تزعجني مشاكل الاسنان او اللثة
12. The condition of my teeth and gums is an important part of my overall health.	12. حالة أسناني ولثتي جزء مهم من صحتي العامة.
